# The digital orchard: advanced data-driven technologies in apple breeding and genetic modification

**DOI:** 10.3389/fpls.2025.1725617

**Published:** 2026-01-12

**Authors:** Fazeel Abid, Zhao Zhang, Ghulam Farooque, Rana Muhammad Zulqarnain, Jawad Rasheed, Onur Osman, Shtwai Alsubai, Leila Jamel

**Affiliations:** 1 Department of Computer Science and Information Technology, The University of Lahore, Lahore, Pakistan; 2 Key Laboratory of Smart Agriculture System Integration, Ministry of Education, China Agricultural University, Beijing, China; 3 Key Laboratory of Agricultural Information Acquisition Technology, Ministry of Agriculture and Rural Affairs, Beijing, China; 4 Department of Mathematics, Saveetha School of Engineering, SIMATS Thandalam, Chennai, Tamilnadu, India; 5 Department of Computer Engineering, Istanbul Sabahattin Zaim University, Istanbul, Türkiye; 6 Department of Software Engineering, Istanbul Nisantasi University, Istanbul, Türkiye; 7 Research Institute, Istanbul Medipol University, Istanbul, Türkiye; 8 Applied Science Research Center, Applied Science Private University, Amman, Jordan; 9 Department of Electrical and Electronics Engineering, Istanbul Topkapi University, Istanbul, Türkiye; 10 Department of Computer Science, College of Computer Engineering and Sciences in Al-Kharj, Prince Sattam Bin Abdulaziz University, Al-Kharj, Saudi Arabia; 11 Department of Information Systems, College of Computer and Information Sciences, Princess Nourah bint Abdulrahman University, Riyadh, Saudi Arabia

**Keywords:** apple breeding, machine learning (ML), deep learning (DL), CRISPR/Cas9 genome editing, high-throughput phenotyping (HTP), agricultural internet of things (AIoT)

## Abstract

The apple (Malus × domestica), a globally significant perennial fruit crop, faces immense pressure from climate change, evolving pathogens, and consumer demand for novel traits. Also, remains constrained by slow trait selection despite technological advances. Further, the traditional breeding methods are slow and resource-intensive, hampered by the apple’s long juvenile period and high heterozygosity. This systematic literature review (SLR) synthesizes the state of the art in advanced data-driven technologies for accelerating apple breeding and genetic modification. Following the PRISMA-EcoEvo protocol, 47 selected studies were analyzed from databases including Web of Science, Scopus, and PubMed. Our thematic synthesis reveals a paradigm shift towards a “digital breeding” model, characterized by the convergence of three core technological pillars. First, high-throughput phenotyping (HTP), which leverages sensor modalities such as RGB-D, hyperspectral imaging, and LiDAR, is automating the collection of trait data at an unprecedented scale. Second, machine learning (ML) and deep learning (DL) algorithms are being deployed for diverse applications, including cultivar identification with over 96% accuracy, non-destructive quality prediction, and genomic selection, thereby boosting predictive ability for key traits by up to 18%. Third, precise and efficient genome editing, predominantly using Clustered Regularly Interspaced Short Palindromic Repeats (CRISPR)/CRISPR-associated protein 9 (Cas9), is enabling the rapid introduction of desirable traits, such as disease resistance, enhanced shelf life, and improved nutrient uptake. Demonstrated transgene-free editing protocols are accelerating the path to commercialization. We further explore the integration of these pillars through the agricultural internet of things (AIoT) and discuss emerging frontiers, including federated learning for data privacy, explainable AI (XAI) for model transparency, and the implications of recent regulatory frameworks. This review identifies critical research gaps, including the need for standardized open-access datasets and integrated end-to-end system validation. It concludes that the synergistic application of these technologies is poised to revolutionize the speed, precision, and resilience of apple improvement programs worldwide.

## 1 Introduction

The apple (Malus × domestica) is one of the most economically and culturally crucial temperate fruit crops, with a global production exceeding 86 million tons annually. Its genetic improvement is critical for ensuring food security, meeting evolving consumer preferences for quality and novelty, and adapting cultivation to the mounting pressures of climate change, including abiotic stresses and the emergence of new pathogen strains (Jamshidi et al., 2025). However, conventional apple breeding is a notoriously slow, expensive, and laborious endeavour. Key biological constraints include a long juvenile phase (5–12 years from seed to first fruit), high levels of genetic heterozygosity, which complicate the fixation of desired traits, and the large land area required to evaluate thousands of unique progenies over many years (Lee et al., 2024). Comparatively, assessing high-throughput phenotyping and genomic selection for apples is feasible due to digitalization and reducing the long juvenile phase to (5-7) years, as well as predicting significant selection and combination of parents ahead of crossing or mutation.

The last decade has witnessed a digital revolution across agriculture, driven by precipitous drops in the cost of sensing, computation, and genomic sequencing. This has ushered in an era of “Agriculture 4.0,” where data is a primary asset for optimizing production and management. This includes augmenting big data techniques and IoTs with Artificial Intelligence (AI) as a modern infrastructure for precision agriculture, robotic farming, and data-driven apple genomic prediction and breeding, transforming traditional ways into a digitally integrated computational process.

For plant breeding, this revolution offers a powerful toolkit to overcome long-standing bottlenecks. Advanced data-driven technologies, spanning artificial intelligence (AI), the internet of things (IoT), and precision genome engineering, promise to transform the breeding pipeline from a lengthy, sequential process into a highly parallelized, predictive, and data-intensive science (Mansoor et al., 2025). This systematic literature review (SLR) aims to comprehensively survey, synthesize, and critically evaluate peer-reviewed research on the application of advanced technologies in apple breeding and genetic modification, using PRISMA-EcoEvo rather than PRISMA-2020, with a focus on ecological and evolutionary factors, specifically genotype × environment interactions and long-term adaptability. The scope of this review encompasses:

(1) The use of sensor-based systems for rapid, non-destructive, and scalable measurement of plant traits. (2) The application of AI algorithms for tasks such as cultivar identification, disease detection, yield prediction, and enhancing genomic selection models. (3) The targeted modification of the apple genome, primarily using CRISPR-based systems, to introduce valuable traits. (4). The deployment of the Agricultural Internet of Things (AIoT) and sensor networks to monitor the orchard environment and inform breeding decisions. (5) An exploration of next-generation technologies like federated learning (FL), transfer learning (TL), explainable AI (XAI), and their potential impact on apple breeding. By systematically mapping the current landscape, this work identifies key achievements, persistent research gaps, and future directions. It provides a critical resource for researchers, breeders, and technologists, highlighting the synergistic potential of these tools and techniques to accelerate the development of next-generation apple cultivars that are more resilient, nutritious, and sustainable.

## 2 Methodology

This review is conducted and reported in accordance with the Preferred Reporting Items for Systematic Reviews and Meta-Analyses (PRISMA) guidelines due to substantial heterogeneity across multiple dimensions. Also, different technologies are considered, including high-throughput phenotyping sensors, machine learning (ML) and deep learning (DL), CRISPR genome editing, and IoT networks, along with distinct experimental statistical techniques. Given the focus on a specific taxon within an ecological and agricultural context, we adapted our framework using the PRISMA-Eco Evo extension, which guides reporting on various factors, including genetic background and environmental conditions, and includes variables critical to plant breeding studies (O’Dea et al., 2021). A comprehensive search of peer-reviewed literature was conducted between 2018 and 2025 across multiple electronic databases to ensure broad coverage of research in agricultural, biological, and computer sciences, including Web of Science, Scopus, PubMed, IEEE Xplore, and AGRICOLA. The search query was constructed using a Boolean matrix approach, combining keywords related to the target crop with terms for the technologies of interest. Minor variations of this string were adapted for the specific syntax of each database. Additionally, the reference lists of key review articles and included studies were manually screened to identify any further relevant publications (i.e., snowballing). A representative search string is as follows:

(“Malus domestica” OR “apple” OR “apples”) AND (breeding OR cultivar* OR “genetic improvement” OR “genetic modification” OR “genomic selection” OR “gene editing”) AND (“machine learning” OR “deep learning” OR “artificial intelligence” OR “convolutional neural network” OR “CNN” OR “phenotyping” OR “phenomics” OR “CRISPR” OR “Cas9” OR “IoT” OR “Internet of Things” OR “sensor network” OR “AIoT”).

Studies were selected for inclusion based on a two-stage screening process. The criteria were pre-defined to ensure objectivity and relevance to the research question. The screening process was managed using the systematic review software Rayyan. In the first stage, two independent reviewers screened the titles and abstracts of all retrieved records against the eligibility criteria. In the second stage, the full texts of the potentially relevant articles were retrieved and assessed for final inclusion. For screening of title and abstract, Cohen’s κ value is 0.85 (95% CI: 0.81-0.89), and for full-text assessment, Cohen’s κ = 0.92 (95% CI: 0.87-0.96) is considered. A standardized data extraction form was developed and piloted. For each included study, the following information was extracted: first author, year of publication, journal, geographic location of the study, breeding objective(s), technology/methodology employed, apple cultivar(s) or germplasm used, key quantitative results (e.g., model accuracy, editing efficiency, prediction ability), and principal conclusions. This structured approach facilitates thematic synthesis and comparative analysis.

**Figure 1:**
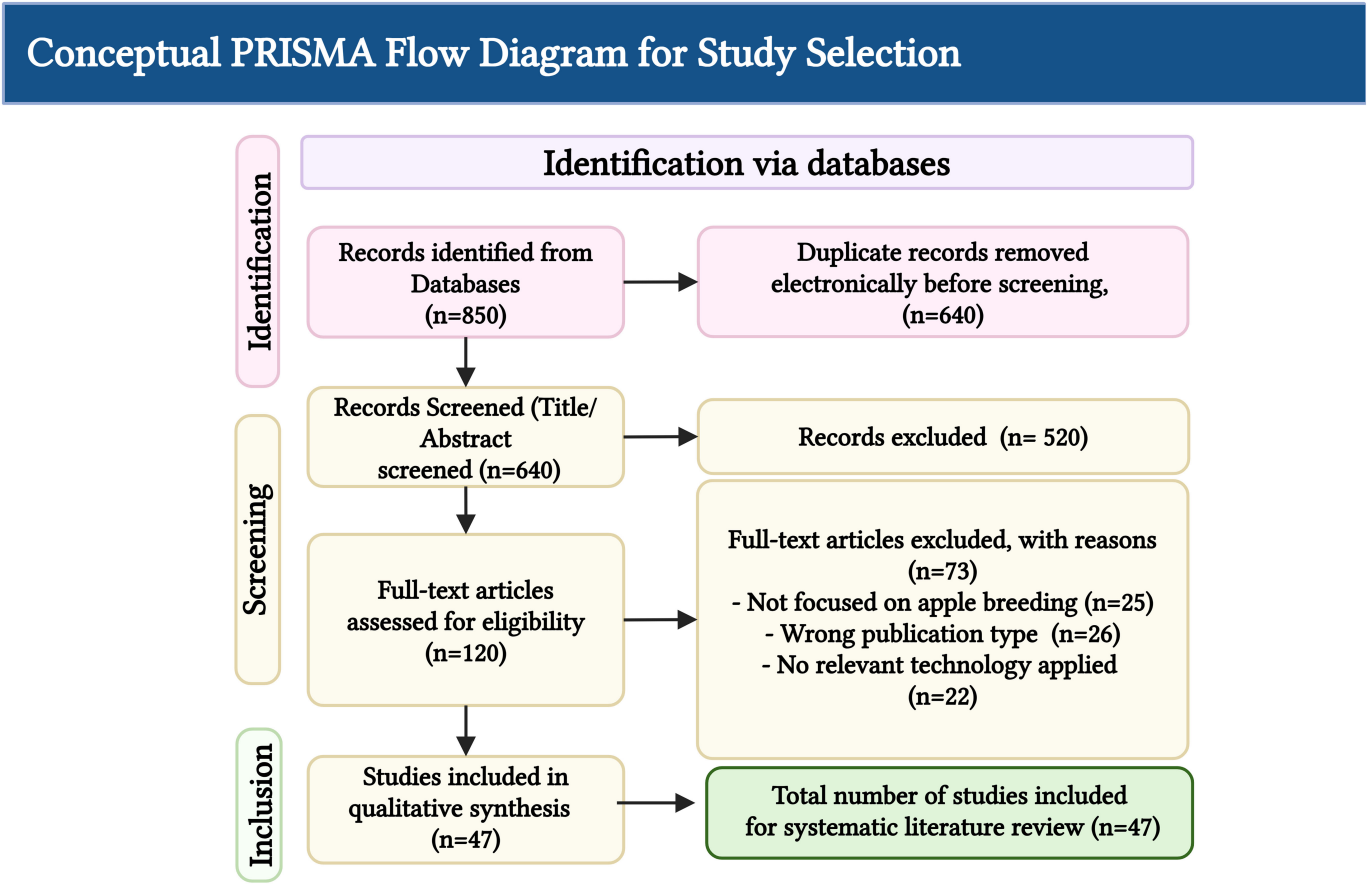
Conceptual PRISMA flow diagram for study selection from 2018 to 2025.

The methodological quality and risk of bias of the included studies were assessed using a checklist adapted from established tools, such as the ROBIS (Risk of Bias in Systematic Reviews) tool, and criteria relevant to experimental research in agricultural biotechnology (O’Dea et al., 2021). Factors considered included the clarity of the research objectives, transparency of the methodology, appropriateness of the statistical analysis, and validity of the conclusions. Following data extraction and quality appraisal, the findings were synthesized thematically. Instead of a quantitative meta-analysis, which was not feasible due to the heterogeneity of technologies and reported metrics, a narrative synthesis was performed. The results were grouped into logical themes corresponding to the core technological pillars identified in the introduction. The synthesis focuses on summarizing the key applications, performance benchmarks, and the overall contribution of each technology to apple breeding. Tables and figures are used extensively in this work to present the synthesized data in a clear, comparative format. A conceptual PRISMA flow diagram for this process is shown in **Figure 1**, illustrating the hypothetical flow of information through the SLR’s phases, from initial identification to final inclusion.

## 3 Results and thematic synthesis

The systematic search identified 47 peer-reviewed articles that met the inclusion criteria. These included studies that directly address genomic breeding, genetic modification, phenomics, or data-driven methods in apple. Whereas the focus on post-harvest traits or storage physiology, without involving genetic, breeding, or trait development, was discarded as presented in **Table 1**. The thematic synthesis of these studies reveals a rapidly evolving and interconnected technological landscape aimed at accelerating apple improvement. We have structured the results into four primary themes: (1) High-Throughput Phenotyping as the data foundation; (2) Machine and deep learning for prediction and classification; (3) CRISPR-based genome editing for targeted trait development; and (4) The integration of these technologies through AIoT and sensor networks.

**Table 1:** The digital orchard: a systematic review of advanced data-driven technologies in apple (Malus × domestica) breeding and genetic modification (criteria).

Inclusion criteria	Exclusion criteria
Original, peer-reviewed research articles (including systematic reviews and meta-analyses) published between 2018 and 2025.	Non-peer-reviewed sources (e.g., conference abstracts, dissertations, patents, editorials, opinion pieces) and published before 2018
The primary subject of the study is the apple (*Malus × domestica*), including its cultivars, rootstocks, or wild progenitors (e.g., *Malus sieversii*).	Studies focused exclusively on post-harvest storage, food processing, or consumer science without a direct link to breeding traits.
The study explicitly applies one or more of the target technologies: ML, DL, FL, TL, AIoT, sensor networks, HTP, CRISPR-based editing, or genomic selection.	Research where apple is only a minor component of a multi-crop study, with no specific results presented for *Malus*.
The application of the technology is directly related to breeding, genetic analysis, or genetic modification objectives (e.g., trait selection, disease resistance, quality improvement)	Studies on pest biology or orchard management that do not involve plant phenotyping or genetic improvement.

### 3.1 Theme 1: high-throughput phenotyping - the data foundation

The “phenotyping bottleneck”, the difficulty of measuring traits accurately and at scale, has long been a significant constraint on plant breeding. Our review reveals a concerted effort by apple to overcome this bottleneck, utilizing a diverse array of sensor technologies. These HTP systems are moving data collection from slow, laborious, and often destructive manual methods to rapid, non-destructive, and automated pipelines. The primary goal is to generate large-scale, high-resolution trait data to fuel downstream genomic selection and ML models. **Table 2** summarizes the key sensor modalities and their applications in apple breeding.

**Table 2:** Sensor modalities and applications in apple high-throughput phenotyping (2018–2025).

Sensor modality	Platform(s)	Key traits measured	References
RGB Imaging	Handheld devices, ground robots, and drones	Fruit count, size, color; leaf shape/color for cultivar ID; disease lesion quantification (e.g., fire blight, scab)	(Chen et al., 2022; Gill et al., 2022; Taner et al., 2023; Yu et al., 2023; Maß et al., 2024; Ropelewska and Lewandowski, 2024)
RGB-D (Depth) Cameras	Ground robots, stationary systems (e.g., FruitPhenoBox)	3D fruit shape and size; fruit volume estimation; canopy architecture; yield estimation	(Keller et al., 2024; Checola et al., 2025),
Hyperspectral & Multispectral Imaging	Benchtop systems, drones	Internal quality attributes (Soluble Solids Content - SSC, firmness); nutrient status; water stress; pigment concentration (anthocyanins, chlorophyll)	(Jung et al., 2025; Zhu et al., 2025),
Thermal Imaging	Drones, field sensors	Canopy temperature as a proxy for stomatal conductance and water stress; early disease detection	(Gill et al., 2022)
LiDAR (Light Detection and Ranging)	Ground robots, tractors	3D canopy architecture (volume, density, leaf area index); light interception; pruning guidance	(Mansoor et al., 2025)

A key trend is the move from 2D to 3D sensing. For instance, a 2023 study coupled YOLO-based object detection with RGB-D cameras to non-destructively estimate fruit count and size directly in orchard breeding plots, enabling HTP at a scale previously unimaginable (Checola et al., 2025). This was further advanced by Keller et al. (2024), who developed the “FruitPhenoBox,” an automated digital phenotyping platform that generates 573 heritable 3D shape and size traits from fruit images. By linking this rich phenotypic data to a GWAS of 303,000 SNPs, they successfully identified 69 significant genetic markers for fruit shape and size, providing immediate targets for marker-assisted selection and gene editing (Keller et al., 2024).

Similarly, hyperspectral imaging is being used to predict internal quality traits without destroying the fruit. A 2025 study demonstrated that Vis-NIR hyperspectral imaging combined with ML models could non-destructively predict color, firmness, SSC, and aroma compounds with a predictive R² greater than 0.75, a crucial tool for selecting elite individuals in a breeding population (Zhu et al., 2025). These HTP technologies are not just generating more data; they are developing new types of data (3D shape descriptors, spectral signatures) that capture complex traits more holistically, providing a richer foundation for genetic discovery and selection. Lastly, this high dimensionality serves as a predictor of various apple traits, such as flavour, quality, and disease susceptibility, irrespective of genetics and breeding.

### 3.2 Theme 2: machine learning and deep learning for prediction and classification

With the explosion of data from HTP and genomics, ML and DL have become indispensable tools for analysis and prediction. Our review identified three major application areas in apple breeding: (1) cultivar and phenotype classification, (2) integration with genomic selection, and (3) generative modeling for in silico screening.

Cultivar and Phenotype Classification: Early and accurate identification of cultivars is essential for managing germplasm collections and culling undesirable seedlings. Several studies have successfully applied ML/DL to this task. One study used convolutional neural networks (CNNs) on leaf images to differentiate between apple varieties, achieving an accuracy of over 96% (Chen et al., 2022). Another comparison of traditional ML models, such as support vector machines (SVMs) and random forests, achieved an F1-score of 0.93 for classifying 10 commercial cultivars based on fruit features (Taner et al., 2023). More advanced methods use a two-stage YOLOv3 + ResNet pipeline to identify fruit varieties in real-time, directly in the orchard, a critical capability for robotic harvesting and in-field phenotyping (Yu et al., 2023). DL is also proving highly effective for disease phenotyping. Another work developed a fine-tuned EfficientNet-B0 CNN that achieved 99.7% accuracy in classifying leaf diseases, providing a scalable tool for scoring disease resistance in large breeding populations (Ali et al., 2025). **Table 3** provides a comparative overview of ML/DL models applied to various breeding objectives.

**Table 3:** Comparative performance of machine learning (ML) and deep learning (DL) models in advanced digital apple breeding applications.

Breeding objective	ML/DL Model(s)	Input data	Performance metric	Reference
Cultivar Identification	Convolutional Neural Network (CNN)	Leaf Images	>96% Accuracy	(Chen et al., 2022)
Cultivar Identification	Random Forest, SVM, k-NN	Fruit HOG + Color Features	0.93 F1-Score (Random Forest)	(Taner et al., 2023)
Real-time Variety Classification	YOLOv3 + ResNet	Orchard Fruit Images	Real-time inference speed	(Yu et al., 2023)
Leaf Disease Classification	Fine-tuned EfficientNet-B0	Leaf Images (Plant Village)	99.7% Accuracy	(Ali et al., 2025)
Fruit Quality Prediction	CARS-MLR, SPA-MLR	Vis-NIR Hyperspectral Data	R²p > 0.75 for firmness, SSC	(Zhu et al., 2025)
Freeze-dried Quality Screen	Various ML Classifiers	CIELAB color + Texture Features	99–100% Accuracy	(Ropelewska and Lewandowski, 2024)
Genomic Prediction of Firmness	Hybrid Genomic-Phenomic Model	SNP Markers + Hyperspectral Data	18% increase in predictive ability over genomics-only	(Jung et al., 2025)

Integration with Genomic Selection (GS): GS models utilize genome-wide markers to predict the genetic merit of individuals, thereby significantly reducing the breeding cycle. A key finding is that integrating HTP data with genomic data can improve prediction accuracy. A study demonstrated that a hybrid model incorporating hyperspectral reflectance data with SNP markers boosted the predictive ability for fruit firmness by 18% compared to a standard genomics-only model (Jung et al., 2025). A review by Ling et al. highlights that ML-based GS pipelines are now being actively piloted in apple rootstock breeding programs, where long juvenile periods make traditional selection particularly inefficient (Ling et al., 2025).

Generative Modeling: A groundbreaking application is the use of generative AI to predict phenotypes directly from genotypes. A work by Jurado-Ruiz et al. introduced “Geno Drawing,” a variational autoencoder that can generate a realistic image of an apple fruit from a low-depth SNP array (Jurado-Rui et al., 2023). This proof-of-concept demonstrates the potential for in silico screening, where breeders could visually assess the predicted fruit of thousands of seedlings before ever planting them in the field, representing a monumental leap in efficiency.

### 3.3 Theme 3: CRISPR-based genome editing for targeted trait development

While ML and GS accelerate selection, CRISPR-Cas9 and related gene-editing technologies enable direct, precise modification of the apple genome. This review reveals a clear progression from early proof-of-concept studies to sophisticated applications targeting commercially valuable traits. A key focus has been on increasing efficiency and developing methods to generate transgene-free edited plants, which face a more streamlined regulatory path. **Table 4** summarizes the significant progress in this area.

**Table 4:** Applications of CRISPR/Cas9 genome editing in apple.

Target gene(s)	Breeding objective	Key innovation/finding	Editing efficiency	Reference
*MdPDS*	Visual marker (albinism)	Overcoming allele-specific targeting in a heterozygous genome	42%	(Jacobson et al., 2023)
Multiple loci	Disease resistance	Multiplex editing in wild apple (*M. sieversii*)	Not specified	(Zhang et al., 2021)
N/A	Method development	Protocol to eliminate chimeras and accelerate recovery of edited plants	N/A	(Li et al., 2023)
Fire blight susceptibility genes	Disease resistance	Combined CRISPR with FLP/FRT for marker-free edited lines	High efficiency	(Pompili et al., 2020)
*MmPHT1;5*	Nutrient efficiency (Pi uptake)	Transgene-free editing in rootstock callus for low-Pi tolerance	Not specified	(Zhao et al., 2025)
Nuclear genes	Method development	Geminivirus-derived replicons for transient, transgene-free delivery	Not specified	(Negishi et al., 2024)
*MdASG1*	Aroma quality & salt tolerance	Linked fatty-acid pathway to stress tolerance (dual-trait target)	Not specified	(Zhang et al., 2024)
Floral AGAMOUS-like genes	Accelerated breeding	Dual-gRNA system for large deletions to create floral mutants	89%	(Jacobson et al., 2023)
*MdSWEET9b*	Flavor improvement	Knockdown of sugar transporter to clarify sucrose loading	Not specified	(Zhang et al., 2023)
*MdLAC7*	Improved shelf-life/cosmetics	Knock-out of the peel-browning gene	Not specified	(Wang et al., 2023)

Early work focused on establishing protocols, including strategies to target genes effectively in the highly heterozygous apple genome (Jacobson et al., 2023) and methods to efficiently select fully edited plants while eliminating chimaeras (Li et al., 2023). More recent research has shifted to trait-focused editing. For example, researchers have targeted genes to reduce susceptibility to fire blight, a devastating bacterial disease (Gill et al., 2022), and improve phosphorus uptake efficiency in rootstocks (Pompili et al., 2020), enhance flavor by modifying sugar transport (Zhang et al., 2023), and prevent cosmetic browning to improve shelf-life (Wang et al., 2023).

A critical breakthrough is the development of methods for transgene-free editing. A work by Negishi et al. successfully used Gemini virus-derived replicons for the transient delivery of the CRISPR/Cas9 machinery into the ‘Fuji’ cultivar (Negishi et al., 2024). This approach achieves the desired edit without stable integration of foreign DNA, a significant advantage for both regulatory approval and public acceptance. This trend, combined with the ability to perform multiplex editing (targeting multiple genes at once) (Zhang et al., 2021), positions CRISPR as a transformative tool for pyramiding multiple desirable traits into elite apple cultivars in a single generation.

### 3.4 Theme 4: the integrated digital orchard - AIoT and sensor networks

The full power of HTP and ML is realized when phenotypic data is contextualized with environmental data. The AIoT provides the infrastructure for this integration. The studies in this review demonstrate the use of wireless sensor networks (WSNs) to create a high-resolution digital image of the orchard environment. A study deployed a network of soil moisture, micro-climate, and leaf wetness sensors in an apple orchard. The resulting five-year dataset was used to train a decision-support model that predicted apple scab incidence with 89% precision, enabling targeted intervention (Jamshidi et al., 2025). Another study showed that guiding irrigation with data from multi-depth tensiometers could reduce water use by 22% without any yield penalty, a critical finding for breeding programs in water-scarce regions (Tumanyan et al., 2023). Furthermore, researchers are developing low-cost, scalable network solutions, such as a LoRa-based mesh network with nodes capable of monitoring the phenology of over 1500 trees simultaneously (Sahar et al., 2023).

**Figure 2:**
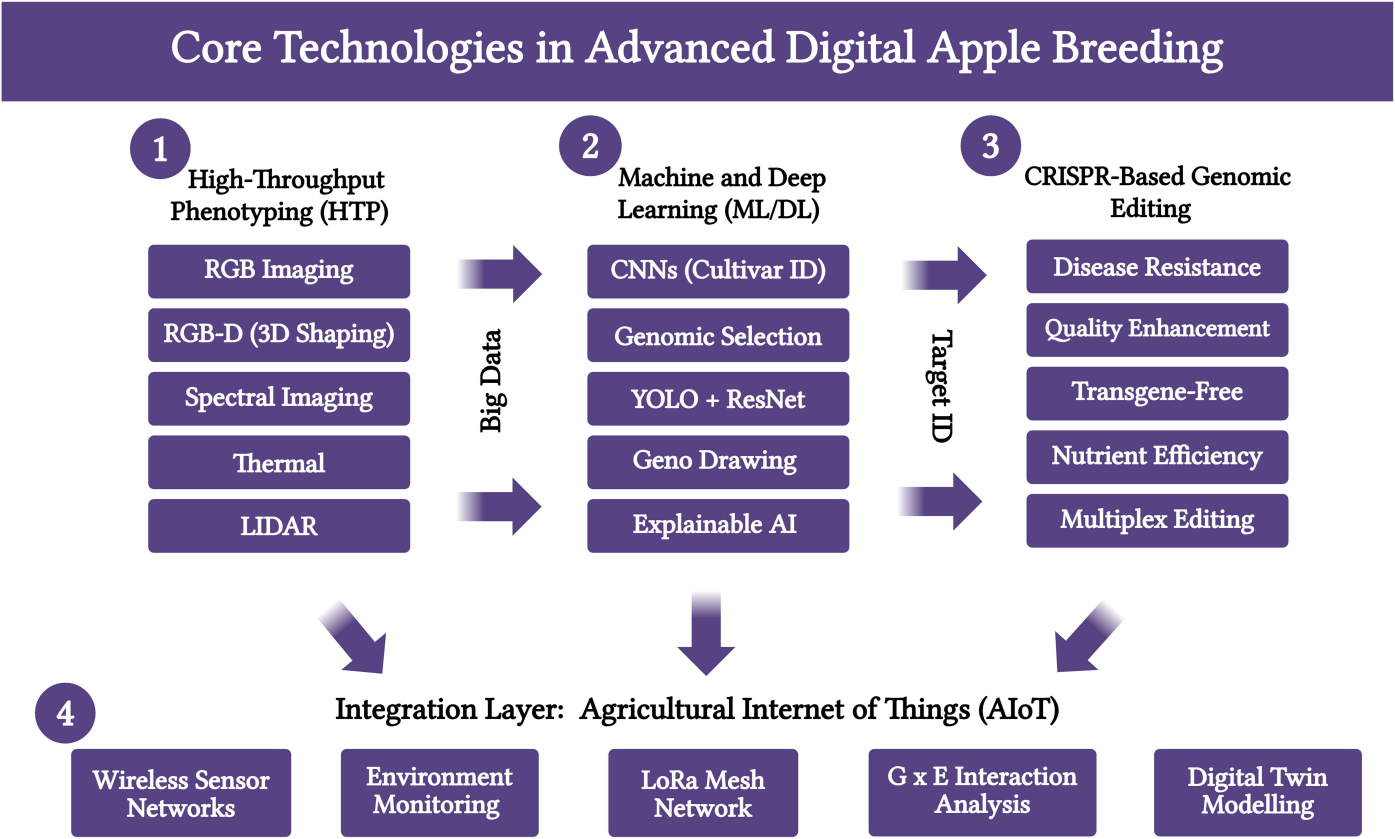
Core technologies and an integration framework driving the advancement of digital apple breeding.

By providing continuous, real-time environmental covariates, these AIoT systems enable breeders to more accurately dissect Genotype × Environment (G × E) interactions. This is crucial for selecting cultivars that are not only high-performing but also stable and resilient across diverse and changing climates. The data streams from AIoT systems form the backbone of a future “digital twin” of the breeding orchard, where every tree’s genetic potential and environmental experience are tracked and modelled. **Figure 2**, review the four themes as core technologies: High-Throughput Phenotyping, Machine/Deep Learning, CRISPR-Based Editing, and Agricultural IoT, integrated to enable advanced digital apple breeding.

## 4 Discussion

The synthesis of research between 2018 and 2025 indicates that apple breeding is at an inflexion point. The disparate technologies of genomics, phenomics, and AI are converging into a cohesive, data-driven ecosystem. This “digital breeding” paradigm is not merely an incremental improvement but a fundamental shift in how new apple cultivars are developed. In this section, we discuss the broader implications of our findings, identify critical research gaps and limitations, and chart a course for future research by exploring emerging technologies and regulatory landscapes.

### 4.1 Synthesis and implications: the digital breeding cycle

**Figure 3:**
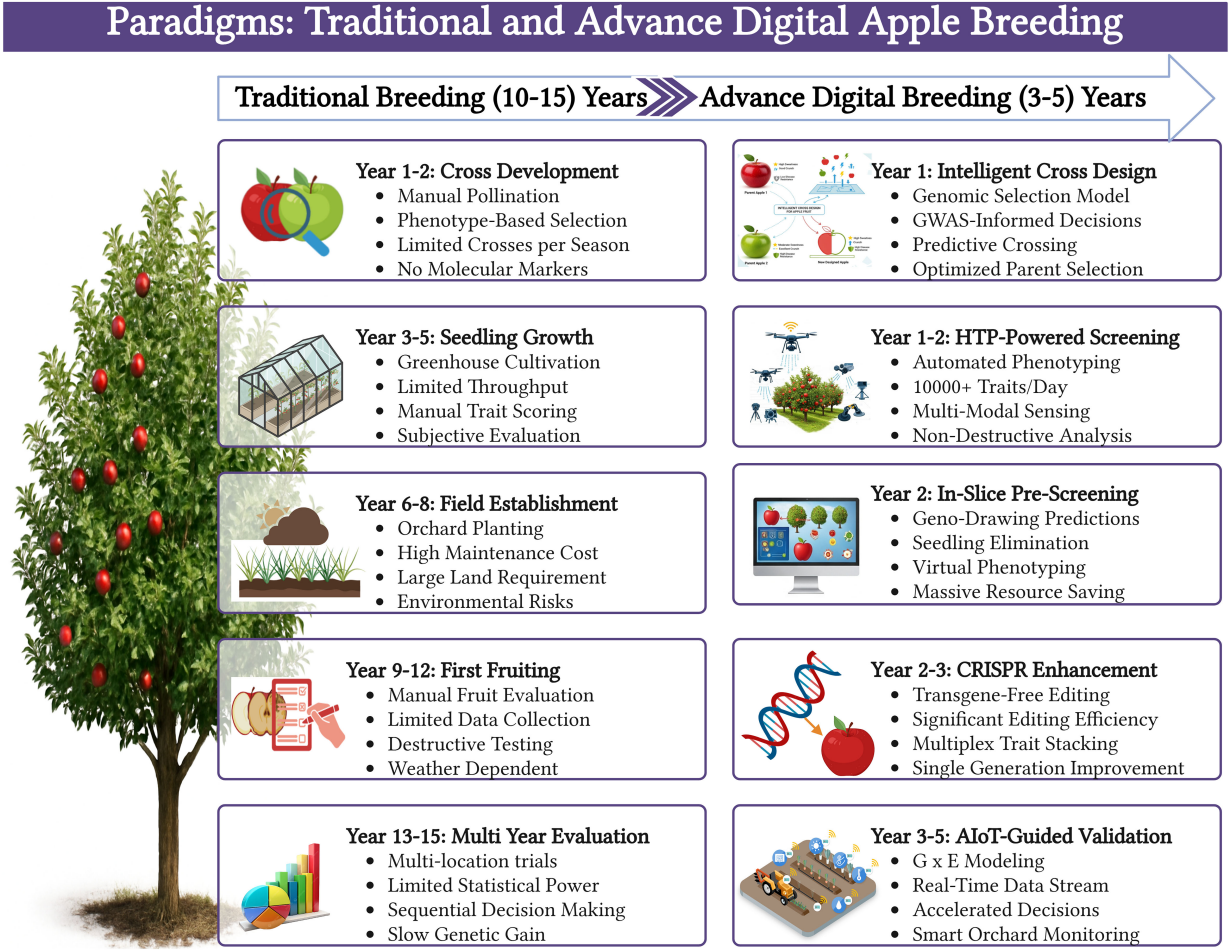
Comparison between traditional apple breeding paradigms, spanning 10–15 years, and advanced digital paradigms, taking 3–5 years, with an emphasis on efficiency, precision, and speed.

The traditional breeding cycle is a long, linear path of crossing, growing, and selecting. The technologies reviewed in this review are reshaping this into a faster, more iterative, and predictive cycle. A future-state digital breeding program can be envisioned as follows: (1) (Genetic Discovery), HTP platforms such as the FruitPhenoBox (Keller et al., 2024) and hyperspectral imagers (Zhu et al., 2025) generate massive, multi-modal phenotypic datasets for a diverse germplasm population. ML-driven GWAS and XAI models (Mohan et al., 2024) then analyze this data to identify novel genes and alleles that control key traits, such as fruit shape, quality, and stress tolerance. (2) (In Silico Pre-Screening), Instead of planting all progeny from a cross, breeders use generative models like GenoDrawing (Jurado-Rui et al., 2023) to predict the fruit phenotype from seedling DNA, culling thousands of undesirable individuals virtually and saving immense time and resources. (3) (Precision Trait Introgression) For high-value traits, breeders use CRISPR to directly edit elite cultivars. Transgene-free delivery methods (Negishi et al., 2024) and marker-free selection systems (Pompili et al., 2020) are employed to introduce desirable alleles for disease resistance or improved quality, enabling the simultaneous pyramiding of multiple traits. (4) (Data-Informed Field Trials), The most promising candidates are planted in AIoT-enabled orchards (Sahar et al., 2023). Real-time environmental and plant status data are continuously collected, enabling precise characterization of G×E interactions and the selection of broadly adapted, resilient cultivars. (5) (Accelerated Evaluation), ML models for disease (Ali et al., 2025) and quality (Ropelewska and Lewandowski, 2024) assessment provide rapid, objective feedback, further shortening the evaluation phase. This integrated approach has the potential to reduce the apple breeding cycle from over a decade to less than five years, while simultaneously increasing genetic gain compared to traditional breeding, as shown in **Figure 3**.

Despite the rapid progress, several challenges, limitations and gaps must be addressed to realize the full potential of digital breeding of apples. While HTP generates vast amounts of data, the field lacks large, standardized, and publicly accessible datasets. This “data drought” hampers the development and benchmarking of robust ML models. Initiatives like the public ERWIAM dataset for fire blight images (Maß et al., 2024) are a crucial step in the right direction, but are still too rare. Secondly, most studies focus on a single component of the digital breeding pipeline (e.g., a new sensor, an ML model, or a CRISPR edit). There is a scarcity of research demonstrating the successful end-to-end integration of these technologies into a cohesive system within an active, large-scale breeding program. Further, the high capital cost of HTP platforms, robotic systems, and AI infrastructure may be prohibitive for public breeding programs and smaller enterprises. Lastly, adequate research is needed on low-cost HTP solutions (Sahar et al., 2023) and the techno-economic viability of these advanced tools to ensure equitable access. Like other fruits, such as Citrus, Grapes and peach, that require computer vision techniques for a canopy assessment, apple, through sensory phenomics, integrates such modelling, despite regulatory challenges in breeding and genetic modification.

### 4.2 Future research directions

The next wave of innovation in apple breeding will likely come from the adoption of emerging data science and computational technologies. Breeding programs are information silos, hesitant to share proprietary germplasm data. Federated Learning (FL) offers a solution by enabling multiple institutions to collaboratively train a shared ML model without exchanging their raw data, preserving privacy and IP (Chorney et al., 2025; Manoj et al., 2025). This could lead to more robust and generalizable models for disease prediction or genomic selection. Similarly, Transfer learning (TL) involves fine-tuning pre-trained models, such as Agri Net (Sahili and Awad, 2022), on smaller, specific datasets. It can dramatically reduce the data and time required to develop effective models for apple-specific tasks.

For breeders to trust and adopt AI-driven selection tools, they must move beyond being “black boxes.” XAI techniques like SHAP and LIME can provide insights into why a model made a particular prediction (e.g., which leaf textures or spectral bands were most indicative of disease) (Mohan et al., 2024; Danilevicz et al., 2025). This transparency is essential for validating models and generating new biological hypotheses (Dublino and Ercolano, 2025). Processing massive HTP data streams in the cloud is slow and costly. Edge computing brings AI inference directly to the sensor or an on-site gateway (David et al., 2024; Padhiary et al., 2024). This enables real-time applications, such as robotic disease scouting or selective spraying, which reduces latency and bandwidth requirements, making AI more practical for in-field deployment (Hoque et al., 2024).

In collaborative breeding efforts, ensuring the provenance and integrity of data is paramount. Blockchain technology can provide a secure, immutable ledger for tracking genomic data, phenotypic measurements, and environmental records across multiple partners, enhancing trust and traceability (Wang et al., 2025; Alobid et al., 2022). While still in its infancy, quantum computing holds long-term potential to solve intractable problems in genomics. Quantum algorithms could one day dramatically accelerate complex tasks, such as genome assembly, haplotype phasing in heterozygous species like apples, or searching for complex multi-locus interactions that are computationally infeasible with classical computers (Kösoglu-Kind et al., 2023; Nałecz-Charkiewicz et al., 2024; Maurizio and Mazzola, 2025). Further, the regulatory environment heavily influences the translation of lab-based innovations to commercial cultivars by providing significant clarity on “foods derived from plants produced using genome editing in (Guidance for Industry: Foods Derived from Plants Produced Using Genome Editing; New FDA Guidance Addresses Regulatory Review of Foods Produced from Genome-Edited Plants). This affirms a risk-based approach, clarifying that many CRISPR-edited plants, particularly those with edits that could be achieved through conventional breeding and that are free of foreign DNA, can proceed through a voluntary pre-market consultation process (New Plant Variety Regulatory Information). This provides a predictable and relatively streamlined path to market for developers of CRISPR-edited apples. Further, AI-driven metabolomics and HTP can be used to efficiently generate the comprehensive data packages required to demonstrate that an edited variety is substantially equivalent to its conventional counterpart. The existence of a public inventory of premarket consultations (Premarket Meetings for Food from Genome-Edited Plants) provides valuable precedents for Apple developers. This favourable regulatory climate, particularly for transgene-free editing techniques (Negishi et al., 2024), is likely to spur further investment and innovation in the application of CRISPR to apple improvement.

## 5 Conclusion

This systematic review confirms that a technological revolution is well underway in apple breeding. The isolated development of high-throughput phenotyping, machine learning, and CRISPR-based gene editing has given way to a period of powerful convergence. The integration of these tools is creating a new digital breeding paradigm capable of overcoming the fundamental biological and logistical barriers that have historically constrained apple improvement. By generating, analyzing, and acting upon data with unprecedented speed and precision, researchers and breeders are poised to develop novel apple cultivars with enhanced climate resilience, disease resistance, nutritional value, and consumer appeal more rapidly. However, to fully realize this potential, the research community must focus on bridging the identified gaps in data sharing, system integration, and economic accessibility. Future success will depend not only on developing more powerful individual technologies but on building open, collaborative, and end-to-end digital ecosystems. By embracing emerging frontiers like federated learning and explainable AI, blockchain, and quantum computing, along with navigating the regulatory landscape with data-rich dossiers, the apple breeding can accelerate the delivery of next-generation apples from the lab to the orchard, ensuring the sustainability and vitality of this critical global crop for decades.
